# Evaluation of the marginal fit and fracture resistance of interim restorations fabricated using different techniques: an in vitro study

**DOI:** 10.1186/s12903-025-05679-y

**Published:** 2025-03-07

**Authors:** Mostafa Elashkar, Yehia Aboushady, Merna Ihab, Mohamed T. El Halawani

**Affiliations:** 1 Department of Conservative Dentistry, Faculty of Dentistry, Alexandria University, Alexandria, Egypt; 2 Department of Prosthodontics, Faculty of Dental Medicine, Alamein International University, Al Alamein City, Egypt; 3 Department of Pediatric Dentistry and Dental Public Health, Faculty of Dentistry, Alexandria University, Alexandria, Egypt

**Keywords:** Interim restoration, Fixed dental prosthesis, 3D-printing, Additive manufacturing, CAD/CAM, Marginal fit, Fracture resistance

## Abstract

**Background:**

Interim restorations are essential for preserving structural integrity and function until the definitive restoration is placed. Their mechanical properties and marginal fit are crucial for clinical performance and are influenced by the fabrication technique and material used.

**Aim:**

The aim of this in vitro study was to investigate the marginal fit and fracture resistance of manually fabricated, computer-aided design and computer-aided manufacturing (CAD/CAM) milled, and CAD/CAM three-dimensionally (3D) printed 3-unit interim fixed dental prostheses (FDPs).

**Materials and methods:**

Sixty-four 3-unit interim FDPs were fabricated on epoxy resin models using different fabrication techniques: manual fabrication with poly methyl methacrylate (PMMA) (*n* = 16), manual fabrication with Bis-acrylic composite resin (*n* = 16), CAD/CAM milling (*n* = 16), and CAD/CAM 3D-printing with a digital light processing (DLP) printer (*n* = 16). The vertical marginal fit of the interim FDPs was evaluated using a stereomicroscope. Following cementation, the specimens were subjected to cyclic loading and then tested for fracture resistance using a universal testing machine. Data were analyzed using one-way ANOVA, and Tukey’s post hoc test was performed to identify statistical differences between the means of independent group pairs.

**Results:**

The smallest marginal gap (31.77 ± 9.0 μm) was observed in the milling group, followed by the 3D-printing group, with no significant difference between the two (*p* = 0.98). Both groups demonstrated significantly smaller marginal gaps compared to the manual fabrication groups (*p* < 0.001). In terms of fracture resistance, the 3D-printing group showed the highest values (1244.46 ± 290.04 N), followed by the milling group, with no significant difference between them (*p* = 0.32). Both groups exhibited significantly higher fracture resistance than the manual fabrication groups (*p* < 0.001).

**Conclusion:**

CAD/CAM 3D-printed and milled interim FDPs demonstrated superior marginal fit and fracture resistance, making them more suitable than conventional techniques, particularly for multi-unit restorations or long-term applications.

## Background

Interim restorations (IRs) play an essential role in the course of fixed prosthodontic treatment. They fulfill several biological and mechanical requirements, maintaining occlusal function and gingival health, they also aid in predicting the final treatment outcome in terms of function, esthetics, and hygiene [1, 2]. Like definitive restorations, adequate marginal fit of IRs is necessary to ensure gingival health and prevent cement microleakage [3]. IRs should also be strong enough to withstand masticatory forces during clinical service, particularly in multi-unit restorations, such as three-unit FDPs, which experience higher occlusal loads. Careful choice of the fabrication technique and material is essential to ensure their strength and long-term clinical success [4].

Materials commonly used for chairside fabrication of IRs are Mono-methacrylates (e.g., poly methyl methacrylate [PMMA]) and Di-methacrylates (e.g., Bis-acrylic composite resin) [1, 2, 5]. PMMAs are relatively inexpensive, but have several drawbacks, including poor wear resistance, significant shrinkage, exotherm during polymerization, and possible pulpal irritation [6, 7]. In contrast, di-methacrylates exhibit lower volatility, reduced shrinkage, minimal exotherm, overall better mechanical properties, and improved marginal adaptation, owing to their large molecular size [1, 7, 8]. Chairside fabrication of IRs presents several limitations, such as extended chair time, operator-dependent quality, and inferior surface texture, strength and fit, due to the incorporation of air bubbles during the mixing and dispensing procedures [4, 9–11].

The current use of digitally fabricated IRs using computer aided design and computer aided manufacturing (CAD/CAM) technology overcame multiple drawbacks of chairside fabrication, allowing for reduced chair time, improved accuracy, and better quality of the resultant restorations [7, 8, 12, 13]. A variety of polymeric materials have been utilized for fabricating CAD/CAM interim restorations, including polymethyl methacrylates, acrylate polymer materials, hybrid composite resins, polyetheretherketone (PEEK), and polylactic acid (PLA), offering distinct properties for various clinical applications [11, 14, 15]. The CAM technology is classified into two main categories: subtractive manufacturing (SM) and additive manufacturing (AM) techniques. SM mills restorations from preprocessed, highly cross-linked PMMA disks, ensuring consistent quality, high accuracy, and superior mechanical properties [4, 9, 10]. However, it generates significant material waste, requires frequent tool replacement, and struggles with producing complex geometries due to milling bur limitations [6, 15–17]. AM, also known as 3D-printing or rapid prototyping, is an economical and sustainable way to introduce the CAD/CAM technology to the dental practice. AM technologies like digital light processing (DLP) build objects by curing liquid resin layer by layer, minimizing material waste and enabling the production of multiple objects simultaneously. AM excels in creating complex geometries and fine details but unlike SM requires post-curing, offers fewer shade options, and may result in less polished surfaces due to anisotropy and the staircase effect [6, 15–17].

Current research presents conflicting evidence regarding the performance of digitally fabricated IRs. While some studies [18–20] suggested that 3D-printed IRs exhibit better marginal fit than milled ones, others [21–23] found the two techniques comparable. Similarly, the evidence on fracture resistance remains inconclusive. Some studies [5, 15, 21] reported that 3D-printed IRs demonstrate superior fracture resistance compared to milled alternatives, while other studies [7, 13, 16] indicated that milled IRs outperform 3D-printed ones in terms of mechanical properties, indicating that further research is needed. Thus, the aim of this study was to compare the marginal fit and fracture resistance of manually fabricated, milled, and 3D-printed 3-unit interim FDPs. The null hypothesis is that different fabrication techniques would not affect the fracture resistance or marginal fit of the IRs.

## Materials and methods

### Study design

This in vitro study evaluated the marginal fit and fracture resistance of four parallel groups of 3-unit interim FDPs fabricated using different techniques: manually fabricated PMMA (Group UF), manually fabricated auto-polymerizing Bis-acrylic resin (Group LT), CAD/CAM milled PMMA (Group SM), and CAD/CAM 3D-printed methacrylate oligomer resin (Group AM) (Table 1). The study was conducted in the Conservative Dentistry Department Laboratory, Faculty of Dentistry, Alexandria University, Alexandria, Egypt.

**Table 1:** Materials, manufacturers and manufacturing methods of the interim FDPs

Material	Group (*n* = 16)	Main components	Manufacturer	Manufacturing method
Unifast III	UF	polymethyl methacrylate (PMMA)	GC Corp., Tokyo, Japan	manually fabricated
Luxatemp Star	LT	Bis-acrylic resin	DMG, Hamburg, Germany	manually fabricated
Ceramill A-Temp	SM	polymethyl methacrylate (PMMA)	AmannGirrbach, AG, Austria	CAD/CAM milled
NextDent C&B MFH	AM	Methacrylate oligomer resin	NextDent, Soesterberg, Netherlands	CAD/CAM 3D-printed (DLP)

### Sample size estimation

The sample size was determined based on the comparison of means reported in previous studies [21, 24], using the following assumptions: confidence level of 95% and study power of 80%. The minimum sample size required was calculated to be 15 specimens per group. To account for potential laboratory processing errors, this was increased to 16 specimens per group. The final sample size was calculated as 4 × 16 = 64 specimens. The calculations were performed using G*Power software (Version 3.1.9.7; Heinrich Heine University Düsseldorf, Germany) [25].

### Master model preparation

**Fig. 1:**
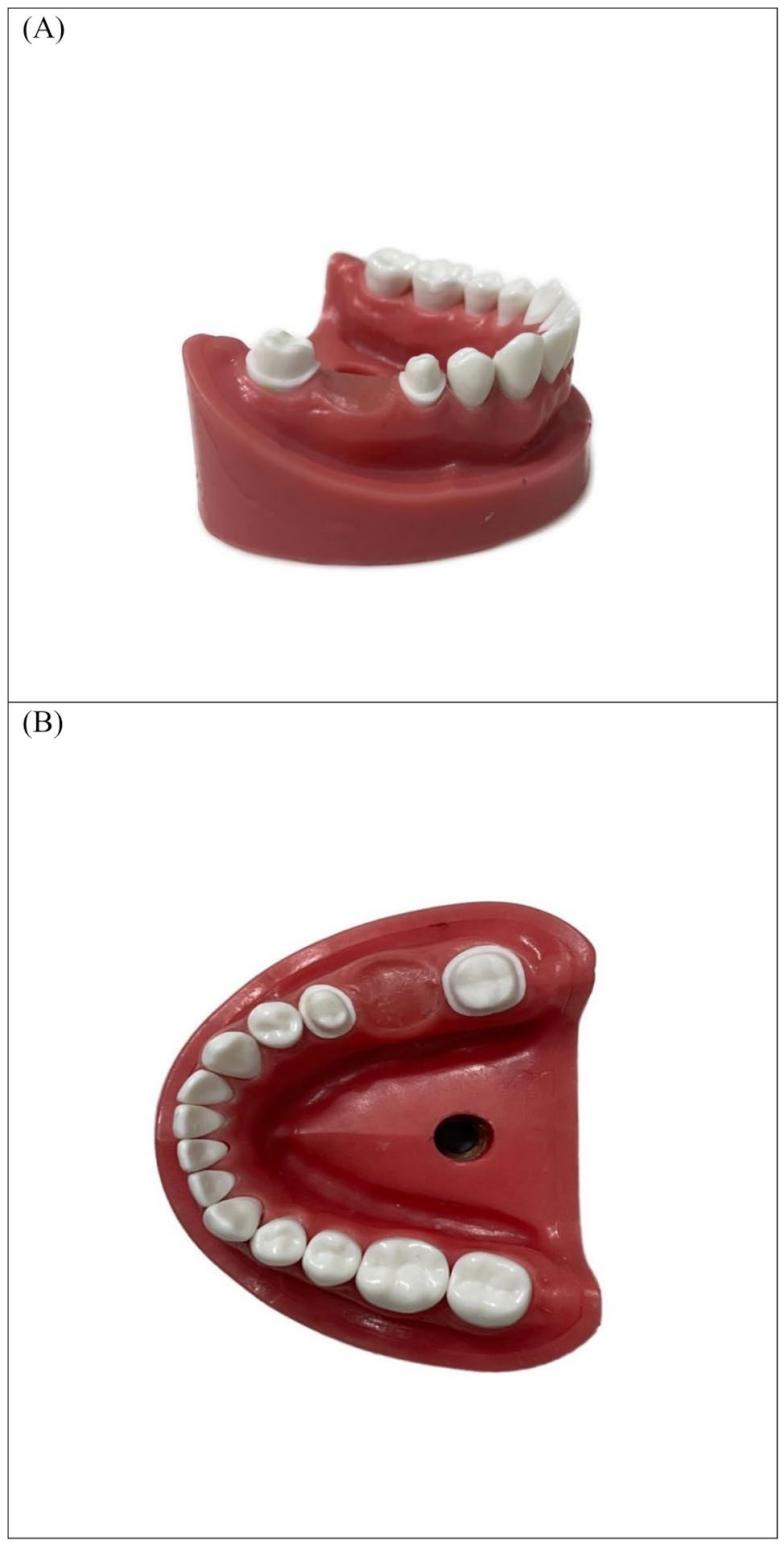
Typodont model with prepared abutments (**A**) Buccal view (**B**) Occlusal view

The mandibular right first molar was removed from a typodont model, followed by the preparation of the mandibular second premolar and second molar for a 3-unit FDP. This preparation included a 1.5-mm occlusal reduction, a 1-mm chamfer finish line positioned 0.5 mm coronal to the cervical line, and a convergence angle of 6 degrees (Fig. 1).

### Scanning and 3D-printing of the master model

**Fig. 2:**
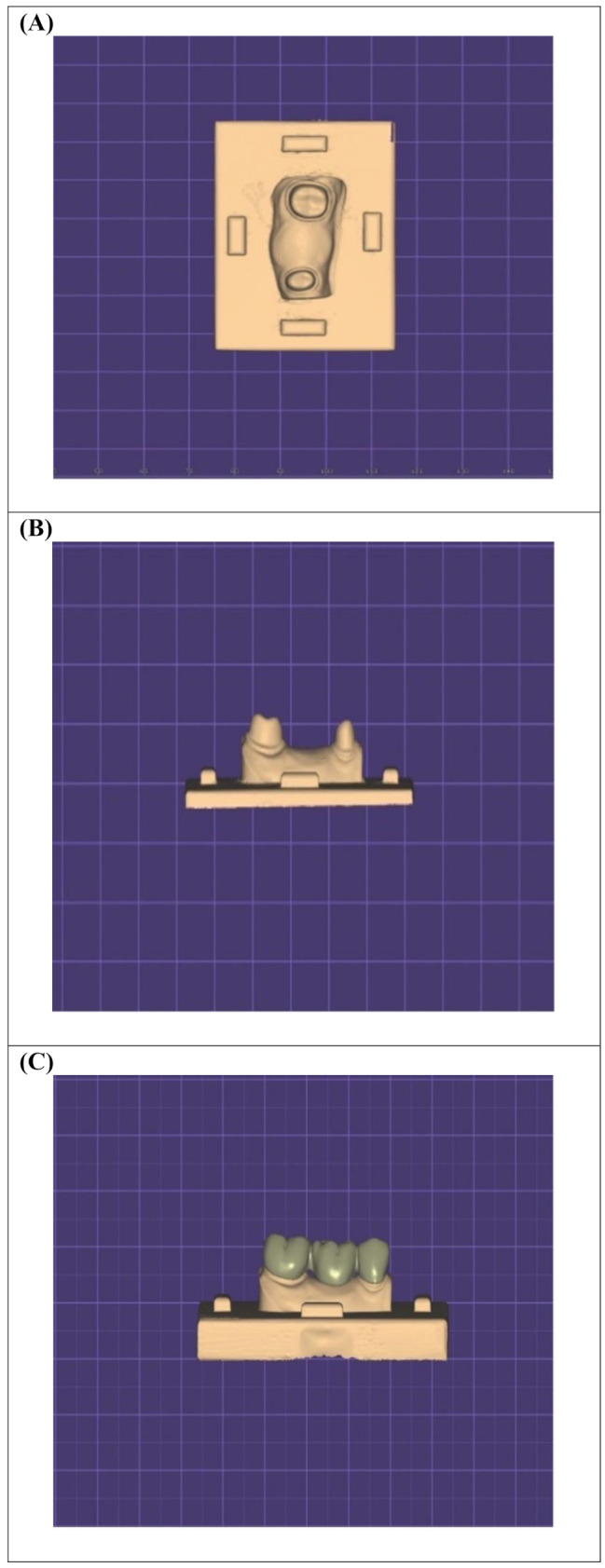
The virtually designed modified master model ready to be printed (**A**) Occlusal view and (**B**) Buccal view. (**C**) Virtual merging of the designed FDP on the modified master model to create the template model

**Fig. 3:**
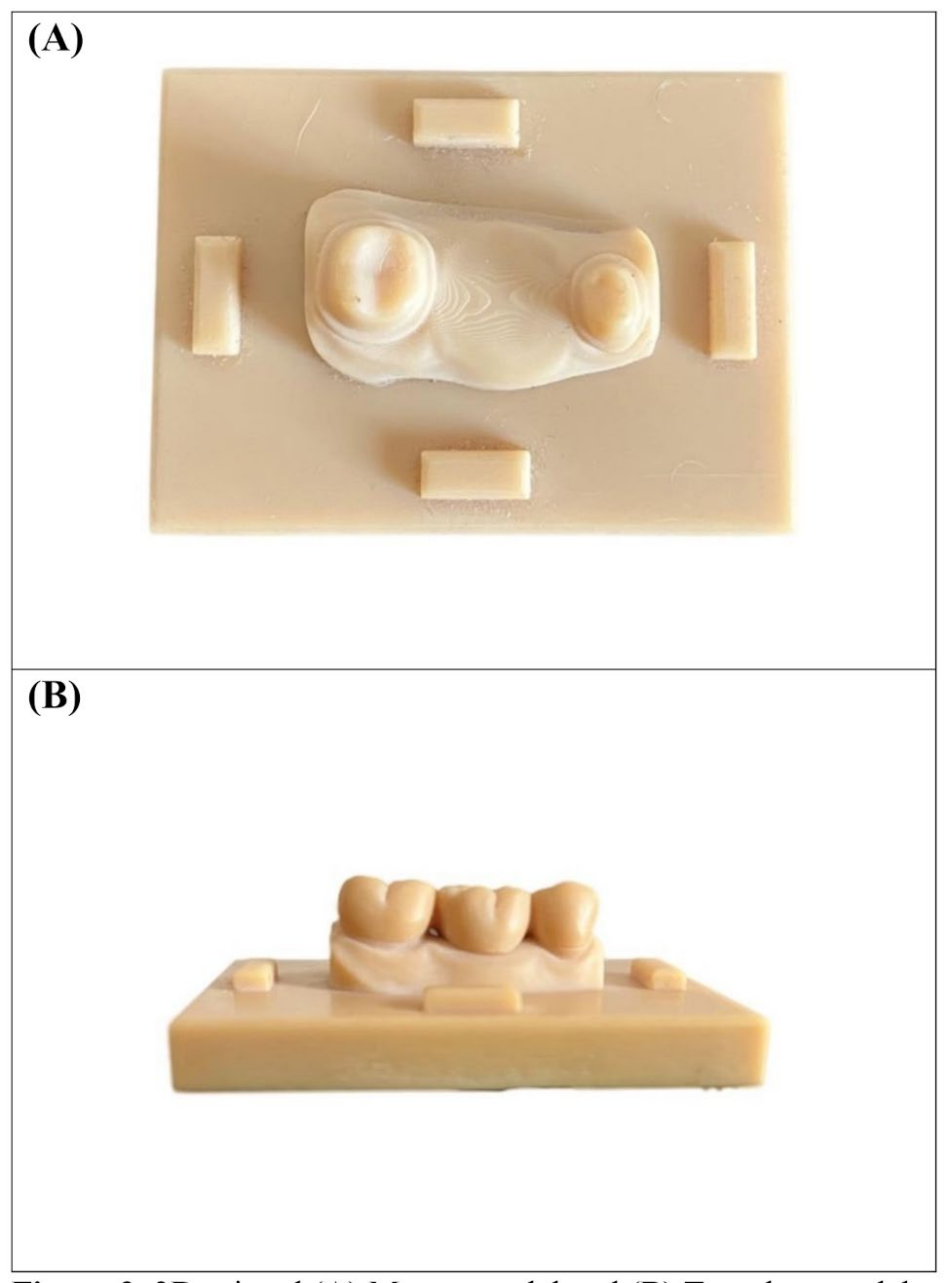
3D-printed (**A**) Master model and (**B**) Template model

The prepared model was digitally scanned using a desktop scanner (Ceramill Map 400; AmannGirrbach, Koblach, Austria). Prior to scanning, the scanner was calibrated following the manufacturer’s guidelines. The standard tessellation language (STL) file of the scanned data was imported into a CAD software (Exocad DentalCAD version 2.4 plovdiv; exocad GmbH, Darmstadt, Germany). The scanned model was trimmed virtually to the area of the prepared teeth. A flat rectangular base part with four rectangular knobs one on each side was designed and merged with the virtually trimmed model to create a master model Fig. 2 (A, B). A full anatomic 3-unit master FDP was designed on the virtual master model, the connectors were set to be 4 mm occluso-gingivally and 3.25 mm bucco-lingually [9], with the cement space specified as 90 μm. The designed master FDP was saved into STL file format to be used for the milling and printing groups, and then was digitally merged on the modified master model, to create a template model as the external surface form for the manual fabrication technique Fig. 2(C), to standardize the fabrication process. The virtually created master model and template model were 3D-printed with model resin material (NextDent Model, NextDent, Soesterberg, Netherlands) on a DLP printer (NextDent 5100, NextDent, Soesterberg, Netherlands) Fig. 3 (A, B).

### Grouping of specimens

The printed master model was then duplicated into 64 identical working models using epoxy resin (RenCast™ CW, UK). The models were then randomly allocated to four experimental groups (*n* = 16 per group), according to the fabrication technique and material.

### Manual fabrication of interim FDPs: groups UF (*n* = 16) and LT (*n* = 16)

A light coating of petroleum jelly (Vaseline, Unilever PLC, LONDON, UK) was applied on the working models to act as a separating medium. FDPs were fabricated by an over-impression technique, where polyvinyl siloxane molds (Elite HD+; Zhermack, Badia Polesine, Roufgo, Italy) using putty consistency, and light body were made over the template model. V-shaped channels were made on the buccal and lingual surfaces of the molds at the pontic area to allow for the escape of excess material and complete seating of the molds. Auto-polymerizing PMMA was mixed with the recommended powder and liquid ratio and loaded into the molds from base to top to prevent inclusion of voids. Components of auto-polymerizing Bis-acrylic resin were mixed using a self-mixing gun and then injected into the molds. Filled impressions were fitted on their corresponding models, guided by the base and the four knobs on the base of the models. After the manufacturer’s recommended setting time, specimens were carefully removed from the molds, and any excess material was trimmed using acrylic resin trimming burs under 5x magnification. Then specimens were polished with SofLex discs (^3M^ ESPE AG, Seefeld, Germany) and a slurry of pumice.

### CAD/CAM milling of interim FDPs: group SM (*n* = 16)

The previously designed STL file of the master FDP was then used to mill the specimens. 16 interim FDPs were wet milled from Ceramill A-Temp (AmannGirrbach, Koblach, Austria) PMMA resin blanks, using a 5-axis milling machine (Sirona inlab MCX5; Dentsply Sirona, Bensheim, Germany). the recommended PMMA bur set (0.5, 1- and 2.5-mm burs) was used. Following milling, specimens were separated from the blank using an acrylic bur and the supporting structures were removed.

### 3D-printing of interim FDPs: group AM (*n* = 16)

**Fig. 4:**
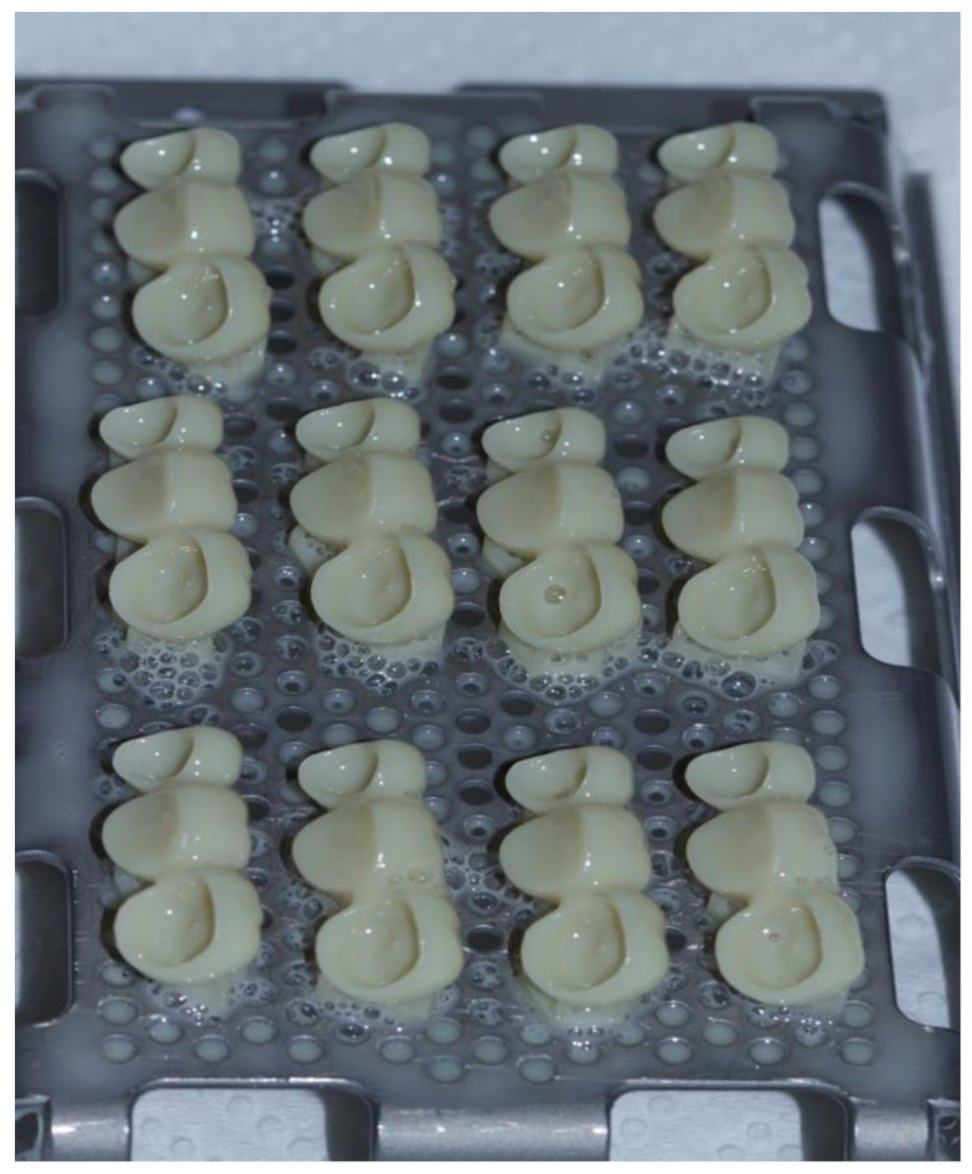
A sample of the 3D-printed specimens on the build plate showing the orientation of printing

The specimens were also printed using the STL file of the previously designed master FDP. A liquid photopolymer resin (NextDent C&B MFH, NextDent, Soesterberg, Netherlands) was mixed for 1 h using a roller stirring device (LC-3D Mixer, NextDent) to ensure thorough blending of the components. Following this, the mixture was used to print 16 interim FDPs with a DLP-based 3D printer (NextDent 5100, NextDent, Soesterberg, Netherlands), with a layer thickness set to 50 μm and a 45-degree build angle configured for the printing process (Fig. 4). To avoid interference with critical areas, such as the margin or intaglio surface, the restoration design was positioned in a way that prevented the supports from connecting to these structures. After printing, the specimens were cleaned with a laboratory wipe and post-cured for 30 min in a curing unit (LC-3D Print Box, 3D Systems, Soesterberg, Netherlands) according to the manufacturer’s specifications to achieve optimal polymer conversion. The supporting structures were then carefully removed. In this study all interim restorations were fabricated by one operator (ME), all measurements were also made by the same operator who was blinded to the restoration materials, to minimize the laboratory errors. All the restorations were also examined for air bubbles, defects, or cracks, and stored in a water bath at 37 ± 1 °C for 24 h before testing.

### Marginal fit evaluation

**Fig. 5:**
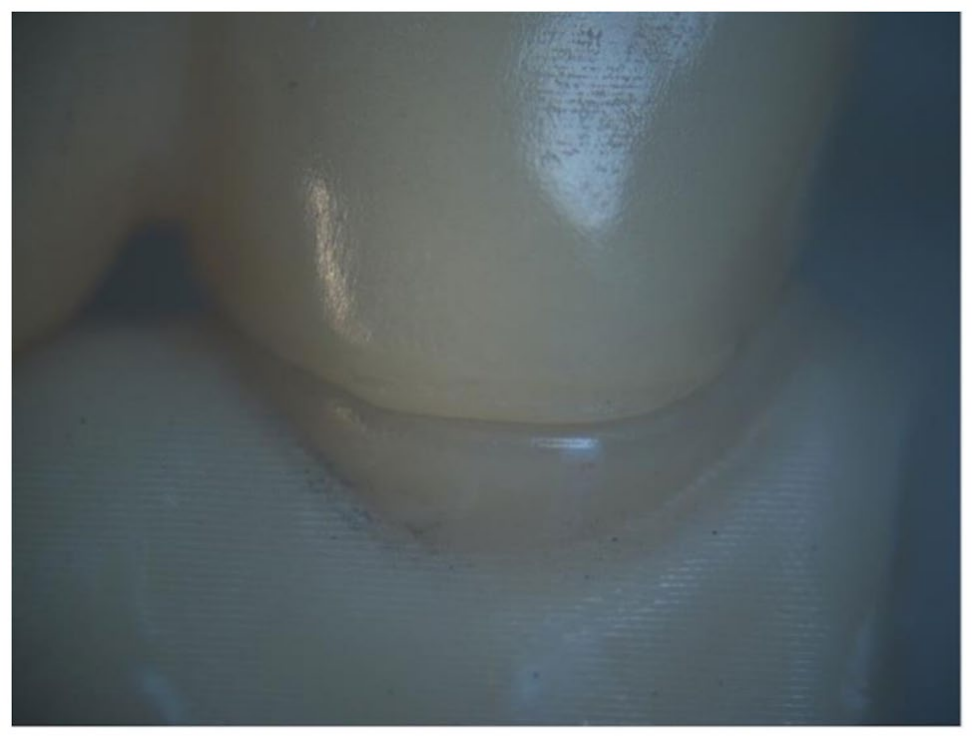
Marginal fit evaluation under stereomicroscope at x25 magnification

Each FDP was seated on its corresponding model and secured with a specially designed holding device, then placed on the platform of a stereomicroscope (B061, Olympus, Japan), which was connected to a digital camera (XCAM1080PHB, Sony, Japan) for detailed imaging. The vertical marginal fit was assessed at a magnification of x25 (Fig. 5). Digital images were captured along the cervical circumference for each retainer and processed with image analysis software (Toup view SW, version 3.7, ToupTek, Hangzhou, China). Measurements were obtained along the margins by drawing a line between the finish line on the die and the retainer’s margin, at 6 sites for each retainer: mesio-buccal, mid-buccal, disto-buccal, mesio-lingual, mid-lingual and disto-lingual, totalling 12 measurements for each FDP. All measurements were recorded in microns and the average gap was calculated for each specimen.

### Cementation of the specimens

Before cementation, each working model was trimmed using an acrylic bur at the pontic site, so that the FDPs weren’t supported by the model during load testing. specimens were then cemented using temporary cement (Cavex BV; Haarlem, Netherlands) under a constant load (5 kg) for 10 min. Excess cement was removed.

### Cyclic loading of the specimens

Specimens were subjected to 60,000 masticatory cycles of vertical load up to 50 N corresponding to the average physiological chewing pressure and 1.7 Hz corresponding to 3 months of function [26] in a custom-made cyclic loading machine (Custom made, Dental Biomaterials Department, Faculty of Dentistry, Alexandria University, Alexandria, Egypt), through a piston with three stainless-steel balls one for each unit (6 mm diameter for the molars, and 5 mm diameter for the premolar).

### Fracture resistance test

**Fig. 6:**
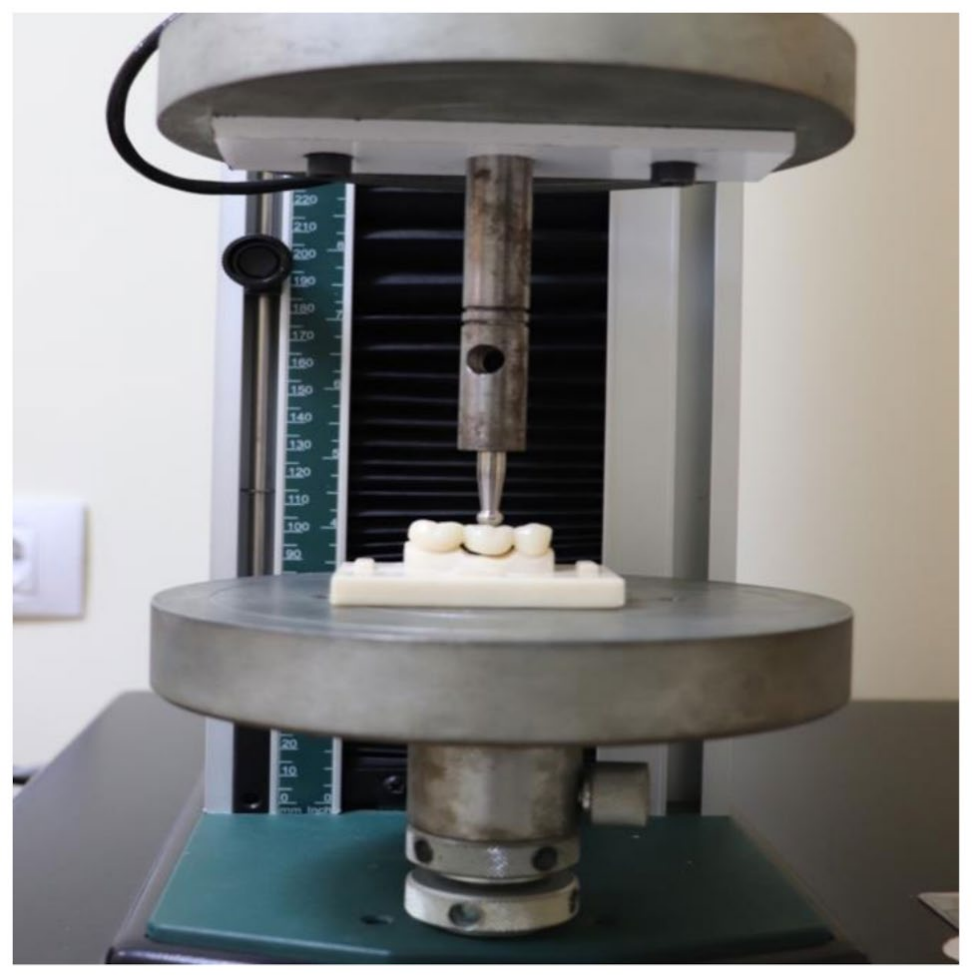
Fracture resistance test in the universal testing machine

Interim FDPs were placed in a universal testing machine (5ST, Tinius Olsen, England), and vertically loaded in the middle of the pontics, at a crosshead speed of 1 mm/min, using a 6 mm diameter stainless-steel spherical indenter until fracture (Fig. 6). Load at failure was recorded in Newtons (N) using the software program (Tinius Olsen 5TH Horizon Software, Surrey, UK).

### Statistical analysis

Visual inspection of data as well as normality testing (Shapiro-wilk and Q-Q plots) were performed. Means and standard deviation (SD) were calculated for all variables, and comparisons of the mean marginal fit and mean fracture resistance between the four study groups were done using one-way ANOVA for normally distributed variables (UF, LT, SM, AM) followed by Tukey’s Post hoc. Significance was inferred at *p* value < 0.05. Data were analyzed using IBM SPSS software for MacBook (Version 29) [27].

## Results

The mean and standard deviation of the marginal gaps for the four study groups are presented in Table 2. The UF group exhibited the largest marginal gap (122.34 ± 23.89 μm), while the SM group demonstrated the smallest (31.77 ± 9.01 μm). Statistical analysis revealed significant differences among the groups (*p* < 0.001). Tukey’s post hoc analysis indicated that the SM and AM groups had significantly lower mean marginal gap values compared to the UF and LT groups (*p* < 0.001). However, no significant difference was found between the SM and AM groups (*p* = 0.98) (Table 3).

**Table 2:** Comparison of marginal fit values among the four study groups

	UF (*n* = 16)	LT (*n* = 16)	SM (*n* = 16)	AM (*n* = 16)
Mean ± SD	122.34 ± 23.89	92.40 ± 10.97	31.77 ± 9.01	33.52 ± 5.14
95% CI	(109.61–135.07)	(86.55–98.24)	(26.97–36.57)	(30.78–36.26)
Min - Max	85–169	77–118	20–55	26–43
F Test (p value)	161.13 (< 0.001 *)			

SD: standard deviation, CI: Confidence Interval, Min: Minimum, Max: Maximum

*Statistically significant at *p* value ≤ 0.05

**Table 3:** Post hoc comparisons of marginal fit values between study groups

Groups	Compared to	Mean Difference	*P* value	95% CI
LT	SM	60.63	< 0.001*	47.42–73.83
	AM	58.88	< 0.001*	45.67–72.08
AM	SM	1.75	0.98	11.45–14.95
UF	LT	29.95	< 0.001*	16.74–43.15
	SM	90.57	< 0.001*	77.36–103.77
	AM	88.82	< 0.001*	75.62–102.03

*Statistically significant at *p* value ≤ 0.05

During dynamic load testing, all specimens across all groups withstood cyclic loading without failure. In static load testing, the AM group demonstrated the highest fracture resistance (1244.46 ± 290.04 N), while the UF group exhibited the lowest (516.93 ± 62.96 N). Significant differences in fracture resistance were identified among the study groups (*p* < 0.001) (Table 4). Tukey’s post hoc analysis revealed that the AM and SM groups had significantly higher fracture resistance compared to the LT and UF groups (*p* < 0.001). However, no significant differences were observed between the SM and AM groups (*p* = 0.32) or between the LT and UF groups (*p* = 0.54) (Table 5).

**Fig. 7:**
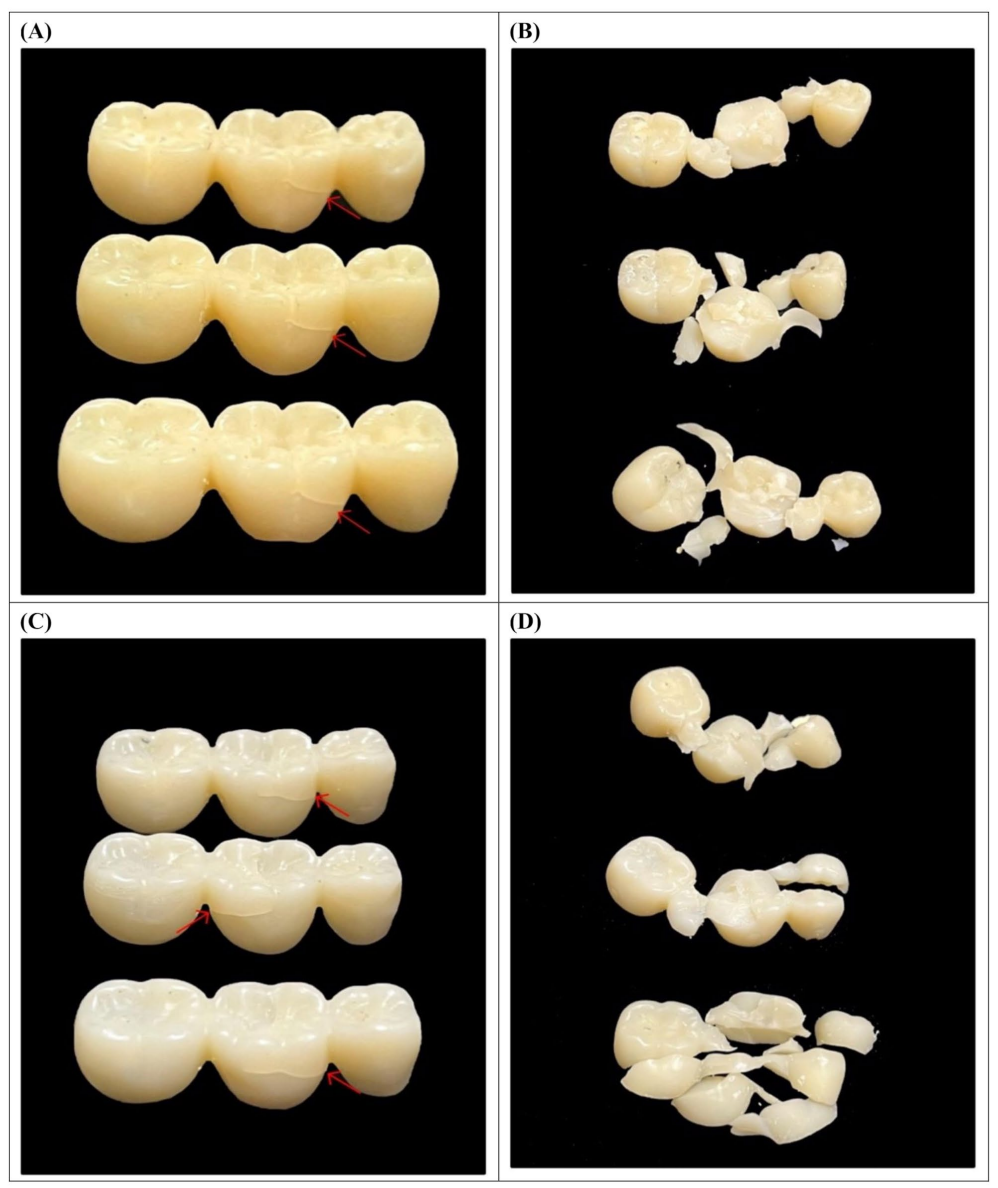
Fracture pattern of tested groups (**A**) group UF showing a crack, (**B**) group LT showing fracture into multiple pieces, (**C**) group SM showing a crack, (**D**) and group AM showing fracture into multiple pieces

Failure modes varied among the groups. Specimens in the UF and SM groups primarily failed with a crack line occurring between the pontic and one of the connectors, whereas specimens in the LT and AM groups fractured into small, multiple pieces (Fig. 7).

**Table 4:** Comparison of fracture resistance values among the four study groups

	UF (*n* = 16)	LT (*n* = 16)	SM (*n* = 16)	AM (*n* = 16)
Mean ± SD	516.93 ± 62.96	597.93 ± 100.19	1141.10 ± 131.36	1244.46 ± 290.04
95% CI	(483.38–550.48)	(544.54–651.32)	(1071.10–1211.10)	(1089.91 − 1399.01)
Min - Max	436–637	449–755	816–1312	1069–2304
*F* Test (*p* value)	76.23 (< 0.001*)			

SD: standard deviation, IQR: Inter- Quartile range, CI: Confidence Interval, Min: Minimum, Max: Maximum. *Statistically significant at *p*-value ≤ 0.05

**Table 5:** Post hoc comparisons of fracture resistance values between study groups CI: confidence interval. *Statistically significant at *p* value ≤ 0.05

Groups	Compared to	Mean Difference	*p* value	95% CI
LT	UF	81.00	0.54	(77.7–239.70)
SM	LT	543.17	< 0.001*	(384.50–701.84)
	UF	624.17	< 0.001*	(465.50–782.85)
AM	LT	646.53	< 0.001*	(487.85–805.20)
	SM	103.36	0.32	(55.31–262.03)
	UF	727.53	< 0.001*	(568.86–886.20)

CI: Confidence Interval. *Statistically significant at p value ≤ 0.05

## Discussion

This study evaluated the impact of different fabrication techniques—conventional methods, CAD/CAM milling, and 3D-printing—on the marginal fit and fracture resistance of 3-unit interim FDPs. All tested groups exhibited vertical marginal gaps within the clinically acceptable threshold of 120 μm [28] except for the conventionally fabricated PMMA group (UF), which slightly exceeded this value (122.34 ± 23.83 μm). Conventionally fabricated interim restorations (UF and LT) demonstrated significantly larger marginal gaps (*p* < 0.05) compared to their digitally fabricated counterparts (SM and AM). Moreover, the digitally fabricated FDPs (AM and SM) exhibited significantly higher fracture resistance (*p* < 0.05) compared to those fabricated using conventional techniques (UF and LT).

Direct evaluation using a stereomicroscope was used for measuring the marginal gap. According to previous literature [29], there is significant variability in the methods used to assess marginal fit, with the direct-view method using a stereomicroscope being one of the most frequently recommended due to its reproducibility and precision. We chose the stereomicroscope owing to its non-invasive, feasible, and practical nature. This approach eliminates the need for specimen sectioning or destruction which was important within the context of our study to allow subsequent cyclic loading and fracture resistance testing. it also minimizes the likelihood of cumulative errors associated with multi-step procedures [18, 29]. However, the technique has limitations, as it can be difficult to distinguish between the tooth structure and the margin, also it can be difficult to measure rounded margins [29]. The vertical marginal discrepancy was assessed following the method described by Holmes and colleagues [30], where the vertical gap was evaluated parallel to the path of the restoration’s draw at multiple points along the margin, between the restoration and its corresponding abutment. Measurements were carried out without cementation, Since the use of cement can result in improper seating of the restoration, which may contribute to an increased marginal misfit [18].

The findings of this study showed that interim restorations fabricated using conventional methods (UF and LT) had significantly larger marginal gaps (*p* < 0.05) than their digitally fabricated counterparts (SM and AM), which agrees with multiple previous studies [12, 21, 22, 31–34]. The inferior marginal fit of conventionally fabricated FDPs may be due to polymerization shrinkage which causes dimensional changes and adversely affects precise fit [12, 31]. Conventional PMMA is composed of monofunctional molecules that have linear structure and low molecular weight, which lack cross-linking. This leads to reduced rigidity and strength, as well as high polymerization shrinkage (∼ 6%) and warpage, which results in marginal gap discrepancies. Bis-acrylic resins are high molecular weight di-methacrylates usually with a variety of multifunctional monomers and inorganic fillers, resulting in a cross-linked structure of improved wear, mechanical properties, and low exothermic temperature. Their chemical structure also allow for lower polymerization shrinkage (1–4%) [35]. Another factor is the manual preparation and trimming of excess material which may negatively affect the fit of the conventionally fabricated restorations [34]. In contrast, the SM group utilized highly crosslinked pre-polymerized blanks, where shrinkage had already occurred during processing before milling. For the AM group, polymerization occurs layer by layer through a UV light source under controlled conditions, minimizing volumetric shrinkage compared to conventional methods, where polymerization happens in a single piece, leading to significantly larger shrinkage [23, 33]. Consequently, digital technologies like CAD/CAM milling and 3D-printing vat polymerization provide superior marginal fit and adaptation due to precise CAM processes and software design [34]. However, a study by Wu et. al [14] reported superior internal fit and absolute marginal discrepancy for conventionally fabricated crowns compared to those made digitally. They attributed these findings to the preset virtual crowns with 60 μm cement space. Despite this, their results for vertical marginal gaps were similar to our study, where both milling and printing groups showed comparable, better results than the conventional Bis-acrylic resin group.

Although milling and 3D-printing methods both use the same CAD process, the slightly larger gap observed in the 3D-printing group of the present study can be attributed to polymerization shrinkage during curing and to dimensional changes during post-processing [19, 36]. Errors in slicing the STL file on the printer’s software can also impact the fit of the restoration [20].

Additionally, printing orientation affects the position and amount of supports generated, with errors potentially occurring from unsupported sections or when supports are placed near the margins [37], also supports placed further away tend to result in rougher margins [38]. Osman and colleagues [39] reported that a 135° printing orientation (equivalent to the 45° used in this study) was optimal for DLP-printed resin crowns. Farag E et al. [40] found that 0° and 45° orientations provided the best marginal fit, as they offered the most self-supporting geometry. printing orientation also affects the appearance of layers created by the 3D printer, changes in layer appearance may impact the form and degree of polymerization shrinkage [41].

The effect of printing layer thickness on marginal fit have shown conflicting results, one study found that layer thickness had no significant impact on marginal fit [42], while another study suggested that a 100-micron layer thickness was superior to 50 microns, attributing this to the accumulation of errors caused by the increased number of layers in the 50-micron thickness [43]. Conversely, another study reported that 3D-printed crowns with a layer thickness between 20 and 50 microns might enhance the margin quality compared to a 100-micron thickness [43].

Other factors influencing 3D-printing accuracy include the material’s chemical composition, printer technology, platform positioning, cleaning, and post curing methods [36, 39, 40, 42, 44, 45]. It should also be noted that the chemical composition of many available 3D printed interim restorative materials has not yet been fully revealed by the manufacturers. Contrary to our findings, some studies observed significantly better fit for 3D-printed restorations compared to milled ones, attributing the larger gap in milled restorations to errors from milling burs during the CAM process [18–20].

Our study also examined the fracture resistance of interim FDPs, with specimens luted using provisional cement to simulate clinical conditions. The mean fracture resistance of the digitally fabricated FDPs (AM and SM) was significantly higher (*p* < 0.05) than that of the conventionally fabricated FDPs (LT and UF), aligning with previous studies [4, 8, 12, 15, 16, 21, 24, 46]. However, other studies [5, 10] reported that the fracture resistance of conventional Bis-acrylic resin crowns was higher than the digitally made ones, especially after thermocycling and 30 days of humid storage. Reeponmaha et al. suggested that Bis-acrylic resin’s higher fracture resistance could be due to its lower water absorption compared to PMMA [5]. In the present study, the AM group demonstrated the highest fracture strength (1244.46 N), followed by the SM group (1141.1 N), consistent with other research [5, 15, 21]. In contrast, some studies found that milled 3-unit interim FDPs had significantly higher fracture resistance than 3D-printed ones [7, 13, 16, 47]. The discrepancy in results could be due to differences in aging protocols. Mayer et al. [48] observed cracks in printed specimens after chewing simulation, which may explain the superior performance of milled restorations. Additionally, some studies indicated that 3D-printed specimens were more susceptible to water storage and humidity, which reduced their fracture load [7, 47].

In this study, the inferior fracture resistance of conventionally fabricated FDPs can be linked to the operator dependent manual fabrication technique and the chemical composition of the materials used [10, 49]. In UF group the manual mixing and dispensing processes can introduce voids, compromising their strength, LT group is supplied in a preloaded cartridge, mixed through an auto-mix delivery system, resulting in a more consistent mix. Air entrapment is still possible though [4, 9–11].

Conventional PMMA exhibited lower strength values compared to Bis-acrylic resin. However, CAD/CAM PMMA performed significantly better than both conventional PMMA and Bis-acrylic resin. This improvement is attributed to the highly polymerized and cross-linked resins used in CAD/CAM PMMA, which are manufactured under high pressure and temperature in a well-controlled industrial environment, resulting in an increased degree of conversion and reduced voids and porosities [5, 10, 49]. Alt et al. [9] reported that Bis-acrylic resin showed significantly higher strength when milled, rather than when manually processed. This confirms that not only the material influences the mechanical properties, but also the fabrication technique.

Several studies [15, 17, 50] investigated the effect of printing orientation on the fracture resistance of 3D-printed interim restorations, finding that a 90° orientation yielded the lowest fracture resistance. The highest fracture loads were observed when specimens were printed diagonally [15, 17] or at 0° [50]. The researchers attributed this to the orientation of the printed layers in relation to the load direction. At 0°, the layers are perpendicular to the load, while at 90°, they are parallel. The 90° alignment places the junctions between layers in the load path, increasing the likelihood of delamination.

Layer thickness is another printing parameter that might have an effect on the mechanical properties of the 3D-printed restorations. A smaller layer thickness have been shown to have higher degree of conversion and possibly greater fracture resistance [24]. However, another study found a 50-micron thickness to have a higher DC than a 25-micron thickness, possibly due to overcuring of the smaller layer thickness [51] indicating that further research is needed.

The superior fracture resistance observed in the AM group was likely due to the layered nature of the 3D-printed structure and the chemical bonding between layers [13]. By following the manufacturer’s recommendations using a 50 μm printing layer thickness, more interfaces were likely formed, leading to a higher degree of polymerization and conversion and reduced residual monomers, which in turn enhanced fracture strength [41]. Also, post-curing processes performed to remove any uncured resin, have been reported to affect the degree of conversion of the object which in turn impacts the precision, accuracy, and overall mechanical properties [51]. In our present study, a strict fabrication process following the manufacturer’s recommendations for the 3D-printing process was employed in a closed system, starting from mixing the resin, printing process and settings till the post-curing process. Consequently, allowing for a more predictable and controllable outcome.

The masticatory forces in the molar region typically range around 350 N but can reach up to 900 N in patients with bruxism [50]. All materials tested in the study demonstrated fracture strength values greater than normal bite forces. However, conventionally fabricated FDPs may not be suitable for patients with bruxism or in extreme oral conditions.

Cyclic loading simulates the repeated mechanical stresses the restorations experience in the oral environment over time, helping in assessing the fatigue behavior of the material and its performance in the clinical environment, and determining how well the material withstands long-term function [52]. In the present study all specimens survived cyclic loading. Two distinct failure modes were observed under static loading: the PMMA groups (UF and SM) failed with only a crack. Cracked restorations are more likely to be retained intraorally, making them safer against patient injury. They can also be repaired and CAD/CAM milled PMMA may be safely used as long-term interim restorations due to its high strength. On the other hand, the LT and AM groups fractured catastrophically into multiple small pieces. This may pose a risk of patient injury and necessitate the remaking of the restorations, requiring additional appointments, increased costs, and patient discomfort. These findings are consistent with previous studies [5, 13, 15, 17, 50] and are attributed to the nature of the materials and their chemical composition. PMMA is more resilient and undergoes significant plastic deformation before fracture, while Bis-acrylic resin and the 3D-printed resin are more brittle and should therefore be used with caution in patients with bruxism.

It’s important to acknowledge the limitations of the current study. These include the inability to replicate the oral environment, the use of only cyclic loading without thermal fatigue testing or water storage, the evaluation of marginal fit before cyclic loading, and the absence of neighboring structures, which can influence stress distribution. Additionally, assessing marginal fit using the direct-view method with a stereomicroscope has limitations, where distinguishing between the tooth structure and the margin can be challenging.

Many 3D-printers are now available commercially in desktop sizes and at much lower costs compared to milling machines [7, 15]. Given the superior performance demonstrated in our study, 3D-printing presents a promising alternative for fabricating interim restorations. However, when selecting an appropriate material for long-term use, factors beyond marginal fit and fracture resistance must be taken into account. Consequently, additional in vitro and clinical studies are necessary to fully assess the viability of 3D-printed interim restorations for long-term application.

## Conclusion

The fabrication technique of interim restorations (IRs) significantly affects their marginal fit and fracture resistance. Digitally fabricated interim FDPs, both additive and subtractive, demonstrated better marginal adaptation and fracture resistance compared to manually fabricated ones. Additionally, additively manufactured interim FDPs show potential as long-term restorations due to their superior marginal fit and fracture strength. However, caution should be exercised when using them for patients with bruxism or parafunctional habits.

## Data Availability

All data generated or analyzed from this study are included in this published article.

## Abbreviations

AM: Additive manufacturing
CAD/CAM: Computer aided design and computer aided manufacturing
CI: Confidence interval
FDP: Fixed dental prosthesis
IR: Interim restoration
Max: Maximum
Min: Minimum
PMMA: Poly methyl methacrylate
SD: Standard deviation
SM: Subtractive manufacturing
